# Greenhouse Evaluation of Clubroot Resistant-*Brassica napus* cv. Mendel and Its Efficacy Concerning Virulence and Soil Inoculum Levels of *Plasmodiophora brassicae*

**DOI:** 10.3390/pathogens10020151

**Published:** 2021-02-02

**Authors:** Nazanin Zamani-Noor, Imke Krohne, Birger Koopmann

**Affiliations:** 1 Julius Kühn-Institute (JKI)—Federal Research Centre for Cultivated Plants, Institute for Plant Protection in Field Crops and Grassland, Messeweg 11-12, 38104 Braunschweig, Germany; 2 Department of Crop Sciences, Division of Plant Pathology and Crop Protection, Georg August University, Grisebachstr. 6, 37077 Göttingen, Germany; ikrohne@hotmail.de (I.K.); bkoopma@gwdg.de (B.K.)

**Keywords:** oilseed rape, clubroot, physiological race, pathotype, resting spore density, resistance overcoming, disease severity index (DSI), area under disease progress curve (AUDPC)

## Abstract

Clubroot resistance of oilseed rape (OSR) cultivars frequently relies on a major resistance gene originating from cv. Mendel. The efficacy of this resistance was studied in greenhouse experiments using two *Plasmodiophora brassicae* isolates, which were either virulent (P1(+)) or avirulent (P1) on Mendel. Seeds of clubroot-susceptible cultivar Visby and clubroot-resistant cultivar Mendel were sown in soil mixtures inoculated with different concentrations of resting spores (10^1^, 10^3^, 10^5^, and 10^7^ resting spores/g soil). Clubroot severity, plant height, shoot and root weight as well as resting spore propagation were assessed for each isolate and cultivar separately at four dates after sowing. The OSR cultivars behaved significantly different in the measured parameters. The threshold of inoculum density to cause disease depended strongly on the virulence of the pathogen and susceptibility of the host plant. In Visby grown in soil infested with P1, clubroot symptoms and increases in root weight and the number of propagated resting spores occurred at inoculum levels of 10^1^ resting spores and higher, whereas Mendel was not affected in soils under the three lowest inoculum densities. In contrast, the P1(+) isolate led to earlier and more severe symptoms, heavier galls, and a significantly higher number of new resting spores in both cultivars.

## 1. Introduction

Clubroot of oilseed rape (*Brassica napus* L.), caused by the obligate soil-borne pathogen *Plasmodiophora brassicae* Woronin, is among one of the most important diseases in oilseed rape production and has reached epidemic levels worldwide [1,2,3,4,5,6]. The clubbed roots, which look like cancerous tumors, lead to reduced water and nutrient uptake, which affect plant growth parameters [7,8]. The disease can significantly reduce seed number and oil content [9,10]. It can cause yield losses between 30 and 50% in fields [11,12] and potentially even cause total yield loss [13].

Particularly, the significance of clubroot disease depends upon the prevalence of virulent pathotypes of the pathogen as well as their affinity or compatibility with the genetic constitution of the host in a given environment [14,15,16,17]. Previous studies in European countries, including the Czech Republic, Denmark, France, Germany, Poland, Sweden, England, and Scotland, have revealed variations in pathotype distribution across different countries [1,2,18,19]. Characterization of European *P. brassicae* isolates on the European Clubroot Differentials (ECD; [20]), and classification by the differential hosts according to Williams [21] or Somé and co-workers [22], showed that pathotypes ECD 16/31/31 and 16/14/31; 4, 6, and 7; and P1 and P3, respectively, are predominant in central Europe [23]. Additionally, several *P. brassicae* isolates of P1 and P3, briefly named P1(+) and P3(+), were found to be moderately or highly virulent on currently available clubroot-resistant oilseed rape cultivars [1,2,18,19,24].

Once *P. brassicae* becomes established in a field, it rapidly builds up resting spores inside roots of susceptible crops, and these can remain in the soil for more than 15 years [12]. Besides the susceptible oilseed rape crop, oilseed rape volunteer seedlings as well as other Brassica crops and weeds can play a critical role in the maintenance of resting spores in infested fields. As such, early destruction of oilseed rape plants and volunteers in clubroot-infested fields has been shown to limit the increase of *P. brassicae* resting spore numbers in the soil [25,26,27,28,29].

Clubroot infections can occur under a low inoculum load of 10^1^ resting spores per g soil [30], though more severe symptoms and higher yield losses occur at resting spore levels greater than 10^3^ spores per gram soil, which is considered the threshold of inoculum density to cause disease in susceptible plants [30,31]. Hwang and co-workers [32] have studied the effect of inoculum density by differentially diluting heavily infested field soil with pathogen-free soilless potting mix. They found that, with the increasing inoculum density, clubroot severity increased and plant height and seed yield decreased. Additionally, the effect of inoculum density of one *P. brassicae* isolate on disease severity and gall formation was also recorded for a new Canadian virulent isolate on a universally susceptible Chinese cabbage cultivar, a susceptible spring oilseed rape cultivar, and 10 resistant canola genotypes [33]. Although root galls were observed at an inoculum density of 10^3^ spores per mL of soil, clear differentiation of susceptible and resistant reactions among spring canola cultivars/lines was not observed until the inoculum density reached 10^5^ spores per mL. At a spore density of 10^6^ spores per mL and above, all spring canola cultivars/lines developed susceptible reactions, though there was some differentiation in the degree of reaction observed [33].

Principally, clubroot disease has been efficiently managed by the use of resistant genotypes. The first clubroot resistance oilseed rape cultivar, *Brassica napus* cv. Mendel, was released in 2006, showing resistance to a number of *P. brassicae* isolates. This cultivar exhibits a race-specific type of clubroot resistance. To date, several clubroot-resistant oilseed rape cultivars have been developed based on Mendel resistance through pedigree breeding. However, this management option with relying on just single dominant-gene resistance becomes more and more questionable owing to the adaption of the pathogen populations. The *P. brassicae*-pathotype present in field soil might significantly influence the relationship between inoculum threshold level and incidence or severity of clubroot disease. Therefore, a better understanding of the factors influencing this relation may lead to better prediction of the disease progress and help to develop more efficient disease management strategies.

In this light, we evaluated the efficacy of cv. Mendel and compared it with the performance of the susceptible cv. Visby, which has had a high market share in Germany since 2008 (German Plant Variety-Catalogue 2008). The interaction of these cultivars was studied with two isolates of *P. brassicae*, which are representatives of the most common pathotypes in European/German fields, as has been shown by Zamani-Noor (2017). The efficacy of clubroot resistance in oilseed rape cultivars was analyzed with regard to the virulence of *P. brassicae* isolates and soil inoculum densities. We also assessed the propagation potential of the different pathotypes producing new resting spores in susceptible and resistant host plants. Further objectives of this study were to assess (i) the development of clubroot disease, (ii) the propagation rate of resting spores within the roots, and (iii) plant growth parameters (e.g., plant height, shoot and root fresh weight (g)) and describing the phenotypic effects of the infection on host plants.

## 2. Results

### 2.1. Influence of Inoculum Density and Virulence of the Pathogen on Clubroot Development

**Figure 1:**
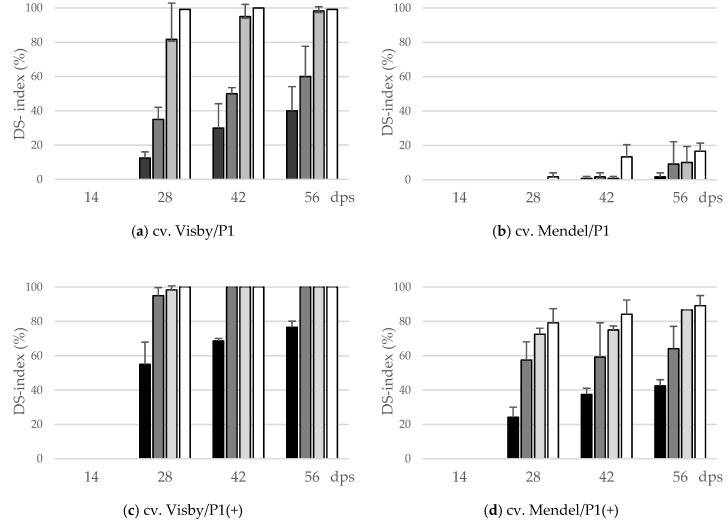
Effect of inoculum density (black bar: 10^1^ resting spores per g soil; grey: 10^3^; light grey: 10^5^; white: 10^7^) and virulence of the *P. brassicae*-pathotypes (P1 vs. P1(+)) on disease severity index (DS-index (%)) of clubroot-susceptible oilseed rape cv. Visby (**a**,**c**) and -resistant cv. Mendel (**b**,**d**). *P. brassicae* P1 isolate was virulent on cv. Visby and avirulent on cv. Mendel, and *P. brassicae* P1(+) isolate was virulent on both cultivars. Seeds were sown in soil mixture inoculated with different concentrations of resting spore inoculum. Disease assessment began at 14 days post sowing (dps), followed by three consecutive measurements at intervals of 14 days. The infection type on each root was visually determined based on a 0–3 scale and disease severity index was calculated from each infection type. Data are pooled across two runs (i.e., repetitions). Error bars indicate standard deviation.

During the first two weeks following sowing, no disease symptoms were observed on plant roots of either cv. Visby or Mendel (Figure 1). Disease symptoms started to develop 14 days post-sowing (dps). The cvs. Visby and Mendel behaved significantly different in all tested parameters in response to inoculum density and pathotype of *P. brassicae*. The interactions of cultivar × inoculum density and cultivar × *P. brassicae* pathotype were highly significant (*p* ≤ 0.05). Disease severity index (DSI) of clubroot susceptible cv. Visby plants grown in *P. brassicae* P1 infested soil increased steadily as inoculum density increased from 10^1^ to 10^7^ resting spores per g soil (Figure 1a). DSI was intermediate and remained below 25% and 50% for inoculum densities of 10^1^ and 10^3^ at 42 and 56 dps, respectively. Symptom expression was progressively more severe, with resting spore loads of 10^5^ and 10^7^ per g soil, in which the clubroot DSI at 28 dps reached 80% and 100%, respectively. At 56 dps, more than half of the plants grown in 10^5^ and 10^7^ resting spores per g soil were dead and exhibited roots that were partly decomposed (Figure 1a). In contrast to the susceptible cultivar, no disease symptoms were observed in clubroot-resistant cv. Mendel plants grown in soil infested with *P. brassicae* P1 until 28 dps, in spite of the high inoculum densities used (Figure 1b). Small galls on root hairs were observed at 42 dps in cv. Mendel for the highest spore density of 10^7^ resting spores per g soil. At 56 dps, clubroot DSI stayed below 20% for this cultivar when grown in soil with 10^5^ and 10^7^ resting spores per g soil (Figure 1b). The mortality rate in cv. Mendel plants, grown in *P. brassicae* P1 infested soil, was zero.

In contrast to *P. brassicae* P1, the highly virulent isolate *P. brassicae* P1(+) had a dramatic effect on disease development and clubroot DSI. In cv. Visby, a DSI of 100% was achieved after 28 dps for all inoculum densities, except 10^1^ resting spores per g soil, in which a DSI of 35% was found (Figure 1c). At 56 dps, the mortality rate was more than 90% when plants were grown in soil with more than 10^1^ resting spores, and most of the roots were partly to completely decomposed. In cv. Mendel, all plants grown in 10^5^ and 10^7^ resting spores per g soil were clubbed, and a disease severity of 80% developed at 28 dps (Figure 1d). Visible clubs with a DSI of 10 and 30% were found even in cv. Mendel grown at lower spore densities of 10^1^ and 10^3^ resting spores per g soil at 28 dps, respectively (Figure 1d). The disease symptoms in cv. Mendel steadily developed as inoculum density increased until 56 dps; however, the plant mortality rate in cv. Mendel remained lower than 15% in all treatments.

The area under disease progress curve (AUDPC) data also show a similar trend. There was a linear relationship between the initial inoculum density and clubroot severity. Both cultivar resistance and pathogen virulence altered the slopes of the regression lines, especially when plants were infected with *P. brassicae* P1. For this isolate, the increase in AUDPC, from the lowest to highest concentrations of inoculum in the soil, increased 5.5 and 14.2 times for cv. Visby and cv. Mendel, respectively, between the lowest and highest concentrations of inoculum in the soil (Table 1), which were higher than 81% for cv. Visby and 92% for cv. Mendel. The AUDPC of cv. Mendel was significantly lower by an average of 94% in all inoculum concentrations in comparison with cv. Visby (Table 1). In contrast, there were minor differences in AUDPC between susceptible and resistant cultivars grown in soil infested with *P. brassicae* P1(+). The highest AUDPC of clubroot was obtained in cv. Visby. In this cultivar, no differences were observed between inoculum concentrations, except 10^1^ resting spores per g soil, which showed significantly lower AUDPC than the other concentrations (Table 1). The AUDPC in cv. Mendel grown in different concentrations of *P. brassicae* P1(+) varied from 624 in soil with 10^1^ resting spores to 2911 in soil with 10^7^ resting spores (Table 1). No significant differences were observed in AUDPC of plants grown in soil with spore density greater than 10^1^.

In all treatments, the AUDPC-value was significantly correlated with the amount of inoculum in the soil (Table 2). The strongest positive correlation between AUDPC and inoculum densities was observed in cv. Visby grown in *P. brassicae* P1 infested soils and then in cv. Mendel grown in soil with *P. brassicae* P1(+). Less strong correlations between AUDPC and inoculum densities were noted in cv. Visby grown in *P. brassicae* P1(+) infested soil and, finally, cv. Mendel grown in *P. brassicae* P1 (Table 2).

### 2.2. Influence of Inoculum Density and Pathogen Virulence on Root Weight and Propagation of Resting Spores

**Figure 2:**
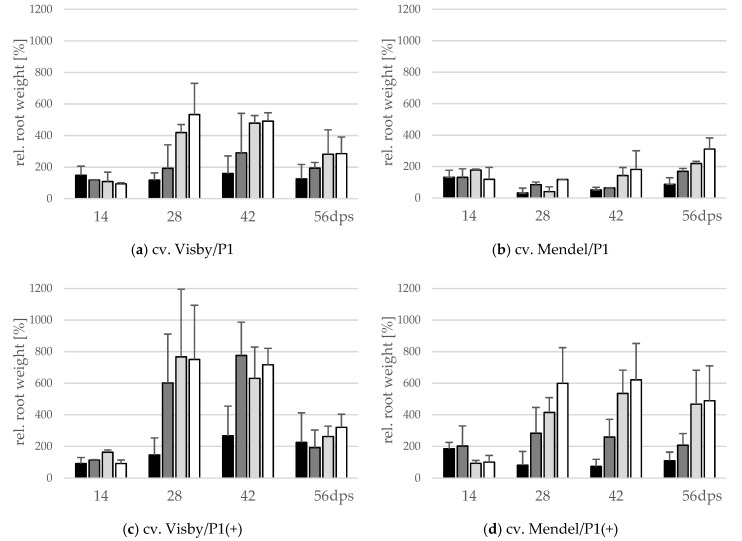
Effect of inoculum density and virulence of the *P. brassicae*-pathotypes on root fresh weight of clubroot-susceptible oilseed rape cv. Visby (**a**,**c**) and -resistant cv. Mendel (**b**,**d**). *P. brassicae* P1 population was virulent on cv. Visby and avirulent on cv. Mendel, and *P. brassicae* P1(+) isolate was virulent on both cultivars. Seeds were sown in soil mixture inoculated with different concentrations of resting spore inoculum (black bar: 10^1^ resting spores per g soil; grey: 10^3^; light grey: 10^5^; white: 10^7^). Measurements of root weight began at 14 days post sowing, followed by three consecutive measurements at intervals of 14 days. At each assessment date, the relative values and mean values of 20 plants (replicates) were calculated. Non-inoculated control plants were set to 100 (bar not shown). Data are pooled across two runs (i.e., repetitions). Error bars indicate standard deviation.

A significant interaction between inoculum density and cultivar and between cultivar and virulence of *P. brassicae* pathotype occurred for fresh root weight (g) and the number of propagated resting spores inside the root. The root fresh weight significantly varied with inoculum density in cv. Visby plants grown in *P. brassicae* P1 infested soil. A slight increase in root fresh weight, reaching up to 1.5 or 2.5 times heavier than the non-inoculated control plants, was observed in soils infested with 10^1^ and 10^3^ resting spores per g soil (Figure 2a), respectively. The root fresh weight increased rapidly up to 4.5 and 5.5 times heavier than in non-inoculated plants at 28 dps in plants grown in 10^5^ and 10^7^ resting spores per g soil, respectively (Figure 2a). After 42 dpi, root fresh weight slightly decreased in all treatments due to root decomposition (Figure 2a). In contrast to cv. Visby, none of the P1 inoculum densities affected the root fresh weight of cv. Mendel when compared with the non-inoculated control plants grown in non-infested soil until 42 dps (Figure 2b). Root fresh weight increased slightly between 42 and 56 dps and this increase was higher as inoculum density increased (Figure 2b). Compared with *P. brassicae* P1, the increase in root growth was significantly greater in both cultivars grown in *P. brassicae* P1(+) infested soils (Figure 2c,d). No significant differences were observed in the root fresh weight of cv. Visby plants grown in inoculum densities of 10^3^ to 10^7^ resting spores of *P. brassicae* P1(+) per g of soil. In comparison with the control plants grown in non-infested soil, the root fresh weight of cv. Visby plants was 6 to 8 times heavier at 28 dps. A continuous increase of weight was then observed until 42 dps. Root fresh weight decreased sharply at 56 dps due to the strong increase in root decomposition (Figure 2c).

Plants of cv. Mendel also behaved differently in response to inoculum density of *P. brassicae* P1(+). Root weight of this cultivar significantly increased in comparison with the non-inoculated controls at all inoculum densities, with the exception of 10^1^ resting spores per g soil (Figure 2d). The heaviest root weight was recorded at 28 dps for 10^7^ resting spores per g soil, although this value was not statistically different from those recorded for 10^3^ and 10^5^ resting spores per g soil (Figure 2d). At this assessment date, the root fresh weight was 3 to 6 times heavier than roots of plants grown in non-infested soils. After 28 dps, no further gain in root fresh weight occurred in plants grown in 10^1^, 10^3^, and 10^7^ resting spores per g soil, just a slight increase was observed in roots of plants grown in 10^5^ resting spores’ soil. Root decomposition was significantly lower in cv. Mendel in comparison with cv. Visby grown in *P. brassicae* P1(+)-infested soil at 56 dps.

**Figure 3:**
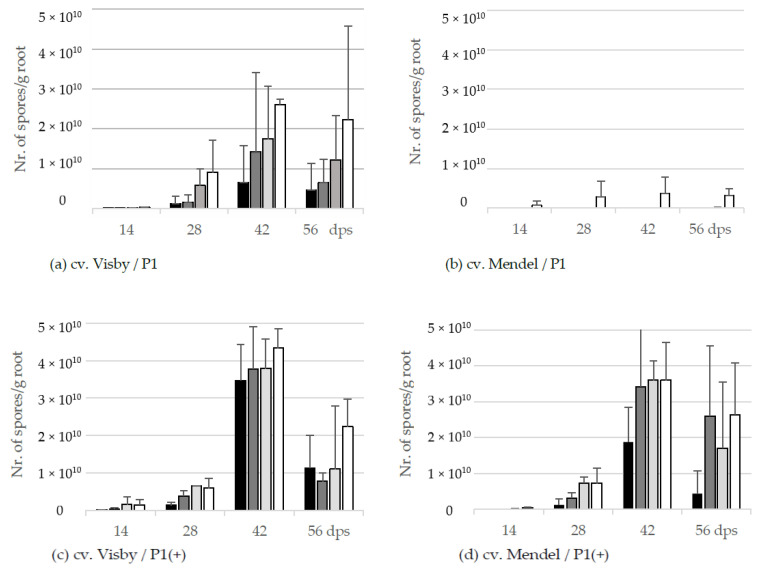
Effect of inoculum density and virulence of the *P. brassicae*-pathotypes on propagation of new resting spore measured by qPCR per g root of clubroot-susceptible oilseed rape cv. Visby (**a**,**c**) and -resistant cv. Mendel (**b**,**d**). *P. brassicae* P1 population was virulent on cv. Visby and avirulent on cv. Mendel, and *P. brassicae* P1(+) isolate was virulent on both cultivars. Seeds were sown in soil mixture inoculated with different concentrations of resting spore inoculum (black bar: 10^1^ resting spores per g soil; grey: 10^3^; light grey: 10^5^; white: 10^7^). Counting of resting spores inside the roots with quantitative real time PCR began at 14 days post sowing, followed by three consecutive measurements at intervals of 14 days. Data are pooled across two runs (i.e., repetitions). Error bars indicate standard deviation.

The resting spore propagation per g root was also affected by inoculum density in cv. Visby plants grown in *P. brassicae* P1 infested soil. Even though no visible disease symptoms were seen at 14 dps, the pathogen DNA-levels inside the root were equivalents of 2.2 × 10^6^ to 2.4 × 10^8^ spores per g root in 10^1^ to 10^7^ inoculum densities, respectively (Figure 3a). At 28 dps, the number of new resting spores per g root ranged from 1.3 × 10^9^ in the roots grown in the lowest inoculum density to 9.0 × 10^9^ in the roots grown in the highest inoculum density. The production of resting spores increased considerably in all treatments until 42 dps, ranging from 6.5 × 10^9^ to 2.6 × 10^10^ in soil infested with 10^1^ and 10^7^ resting spores, respectively (Figure 3a). The number of new resting spores per g root decreased steadily after 42 dps as a result of the root decomposition (Figure 3a).

Despite no disease symptoms being observed in cv. Mendel grown in *P. brassicae* P1 infested soil in lower inoculum densities, very low numbers of new resting spores were detected in roots of this cultivar grown in soils infested with inoculum density lower than 10^5^ resting spores per g soil (Figure 3b). For an inoculum density of 10^7^ resting spores per g soil, the number of new spores increased very slightly from 7.4 × 10^8^ at 14 dps to 3.3 × 10^9^ at 56 dps (Figure 3b).

In contrast to the spore reproduction rate in cv. Visby grown in *P. brassicae* P1 infested soil, no significant differences were observed in the production of new resting spores inside the roots of this cultivar between inoculum densities of the *P. brassicae* P1(+) (Figure 3c). A range of 4.5 × 10^6^ to 1.4 × 10^9^ was observed between the lowest and highest inoculum density at 14 dps, respectively; increasing gently from 1.3 × 10^9^ to 5.9 × 10^9^ at 28 dps; and then more rapidly to 3.5 × 10^10^ and 4.3 × 10^10^ at 42 dps. The number of propagated resting spores inside the roots declined markedly to 1.1 × 10^10^ and 2.2 × 10^10^ at 56 dps as a result of root decomposition and release of resting spores into the soil. The number of propagated resting spores in cv. Mendel grown in *P. brassicae* P1(+) infested soil was lower than cv. Visby grown in the same conditions (Figure 3d). Differences in the number of new resting spores inside the roots of cv. Mendel plants were observed between inoculum densities of 10^1^ with the other densities. The number of new resting spores increased sharply between 28 to 42 dps in plants grown in inoculum densities of 10^3^ to 10^7^ resting spores per g soil of *P. brassicae* P1(+). As roots of cv. Mendel decomposed at slower rates than roots of cv. Visby, the number of new resting spores per g root decreased steadily after 42 dps until 56 dps (Figure 3d).

### 2.3. Influence of Inoculum Density and Virulence of the Pathogen on Shoot Height and Shoot Fresh Weight

**Figure 4:**
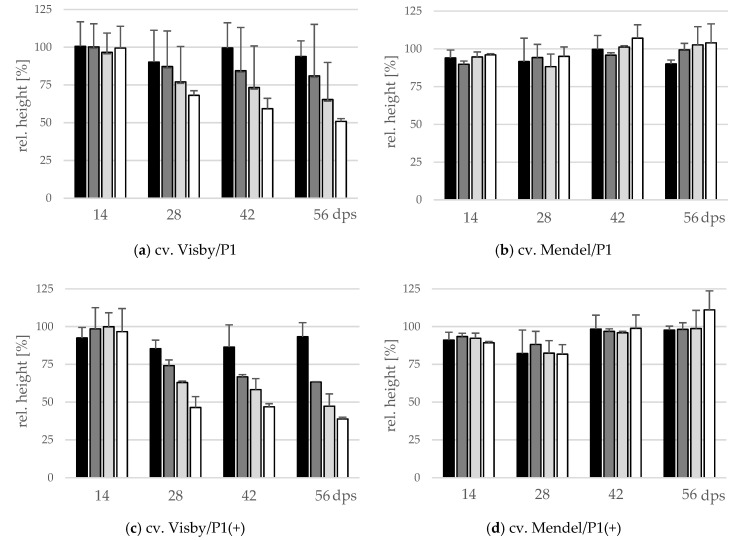
Effect of inoculum density and virulence of the *P. brassicae*-pathotypes on relative shoot height of clubroot-susceptible oilseed rape cv. Visby (**a**,**c**) and -resistant cv. Mendel (**b**,**d**). *P. brassicae* P1 population was virulent on cv. Visby and avirulent on cv. Mendel, and *P. brassicae* P1 (+) isolate was virulent on both cultivars. Seeds were sown in soil mixture inoculated with different concentrations of resting spore inoculum (black bar: 10^1^ resting spores per g soil; grey: 10^3^; light grey: 10^5^; white: 10^7^). Measurements of shoot height began at 14 days post sowing, followed by three consecutive measurements at intervals of 14 days. At each assessment date, the relative values and mean values of 20 plants (replicates) were calculated. Non-inoculated control plants were set to 100 (bar not shown). Data are pooled across two runs (i.e., repetitions). Error bars indicate standard deviation.

**Figure 5:**
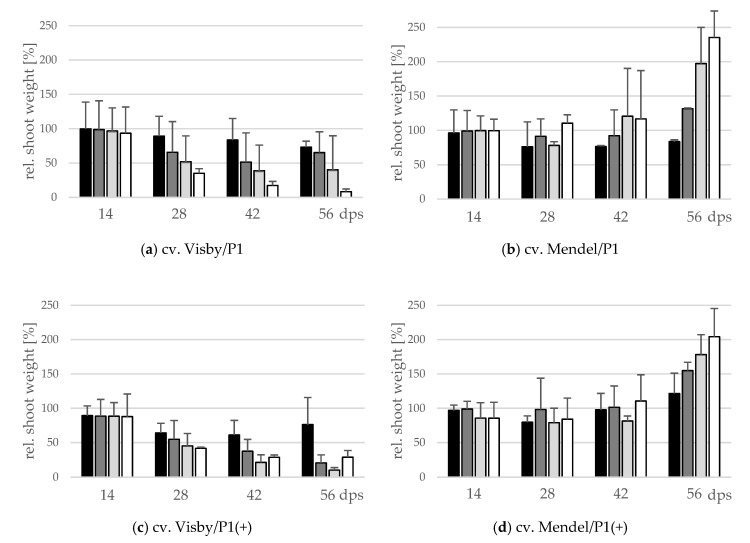
Effect of inoculum density and virulence of the *P. brassicae* pathotypes on relative shoot fresh weight of clubroot-susceptible oilseed rape cv. Visby (**a**,**c**) and -resistant cv. Mendel (**b**,**d**). *P. brassicae* P1 population was virulent on cv. Visby and avirulent on cv. Mendel, and *P. brassicae* P1 (+) isolate was virulent on both cultivars. Seeds were sown in soil mixture inoculated with different concentrations of resting spore inoculum (black bar: 10^1^ resting spores per g soil; grey: 10^3^; light grey: 10^5^; white: 10^7^). Measurements of shoot fresh weight began at 14 days post sowing, followed by three consecutive measurements at intervals of 14 days. At each assessment date, the relative values and mean values of 20 plants (replicates) were calculated. Non-inoculated control plants were set to 100 (bar not shown). Data are pooled across two runs (i.e., repetitions). Error bars indicate standard deviation.

The effect of inoculum density and virulence of *P. brassicae* pathotypes on relative shoot height (cm) and relative fresh shoot weight (g) between the two cultivars was significant (Figure 4 and Figure 5). In clubroot-susceptible cv. Visby, the incremental shoot height (cm) and shoot fresh weight (g) of all plants decreased as inoculum concentration of the pathogen increased (Figure 4a and Figure 5a). This decline was steeper among plants grown in infested soil with *P. brassicae* P1(+). For this isolate, the reduction of the relative plant height occurred earlier and at lower inoculum densities in comparison with the P1-isolate (Figure 4c). No significant differences were found in incremental shoot height (cm) or shoot fresh weight (g) for inoculum densities higher than 10^3^ resting spores per g soil in any cv. Visby plant–pathogen combination. At the last assessment date (56 dps), shoot height and shoot fresh weight were reduced by up to 60% and 90%, respectively. In contrast, in clubroot-resistant cv. Mendel, no differences were found in plant height (cm) between plants grown in infested soils at any sampling time (Figure 4b). In addition, no differences were observed between treatments and plants grown in non-infested soil (Figure 4b). Furthermore, the virulence of the pathogen did not have any influence on plant height in cv. Mendel (Figure 4d). The shoot fresh weight in cv. Mendel remained similar to control plants until 42 dps. Surprisingly, the shoot fresh weight was greater in plants grown in infested soils in comparison with control plants at 56 dps, where this increase was higher as inoculum concentration of the pathogen increased (Figure 5d). The virulence of the *P. brassicae* isolates has no effect on cv. Mendel.

## 3. Discussion

While many strategies have been applied for clubroot management, growing of clubroot-resistant Brassica cultivars is the most effective and sustainable approach to controlling the disease and, currently, all major breeding programs focus on the selection of disease-resistant oilseed rape genotypes as a priority. However, clubroot resistance still primarily relies on single dominant gene-based resistance derived from *Brassica napus* cv. Mendel, often found not to be durable for a long time, and the overcoming of resistance after a few growing seasons in clubroot-infested fields have been reported in different countries [1,2,18,19,24]. Generally, it was found that increasing inoculum amounts translate into increasing values of both disease incidence and severity [26,33]. However, the influence of the pathotype virulence and the susceptibility of the host cultivar on this relationship has not been intensively investigated. In the present study, we investigated for the first time the combined effects of virulence of the pathogen, inoculum density, and host resistance on clubroot resistance efficacy, and thereby potential effects on its durability. The change in development of clubroot disease, the propagation rate of the new resting spores, as well as plant growth parameters in relation to the factors mentioned above were evaluated during the study.

The results clearly showed that the threshold of inoculum density of *P. brassicae* to cause clubroot disease depended significantly on the virulence of the pathogen and the susceptibility of the oilseed rape cultivar. We have seen that an increase in inoculum density of the less virulent isolate of *P. brassicae* (virulent on susceptible and avirulent on resistant cultivar) accelerated the progress of clubroot disease in susceptible oilseed rape cultivars, and that lower inoculum concentrations delayed the expression of clubbing symptoms. By contrast, the presence of resting spores of the higher virulent isolate (virulent on both cultivars) led to symptoms at significantly lower inoculum densities. Development of severe clubroot disease in susceptible oilseed rape cultivar required a much lower inoculum density of the virulent pathotype (*P. brassicae* P1(+)) compared with that of the less virulent one (*P. brassicae* P1) (Figure 1). While more than 10^5^ resting spores per g soil of the less virulent pathotype was required to attain the highest amount of disease in the susceptible oilseed rape cultivar (DSI = 100), in the resistant cultivar, 10^5^ resting spores per g soil were required to produce 5% disease severity. Eventually, a maximum disease severity of 18% was observed in resistant cultivar grown in 10^7^ resting spores per g of soil of *P. brassicae* P1, the highest inoculum density of the pathogen used in this study. 

These results suggest that plant exposure to higher inoculum concentrations could increase infection rates in resistant oilseed rape cultivars. It should be stressed, however, that field isolates were used here as inoculum, and it is possible, on one hand, that as the spore densities in the soil increased, the number of high virulent spores within this population increased too. On the other hand, low levels of high virulent isolates have existed within the population of *P. brassicae* for some years, and the population of high virulent isolates increased because of the selection pressure exerted by extensive use of clubroot-resistant cultivars. Our results confirm previous work [33] showing that the severity of disease reaction, root hair infection rates, and the amount of *P. brassicae* DNA present in each canola genotype varied depending on the strain of the pathogen. Similarly, an earlier study demonstrated strong positive correlations between the amount of *P. brassicae* DNA and the level of root hair infection, where this relationship was stronger for the resistant cultivar than for the susceptible one [9]. Furthermore, we have found that, at equal inoculum densities, clubroot symptoms in susceptible cultivar caused by the high virulent pathotype develop earlier and more rapidly compared with those caused by the less virulent isolate. For the most severe clubroot-conducive, cv. Visby/*P. brassicae* P1(+) combination, the highest disease severity index of 95% was attained with 10^3^ resting spores per g soil at 28 dps, where additional inoculum beyond this level did not result in a significantly higher disease incidence and severity. Conversely, 10^7^ resting spores per g soil of the same pathotype was needed to attain a comparable level of disease in the resistant oilseed rape cv. Mendel.

Present results have also demonstrated that plant genotype, virulence of the pathogen, and inoculum density had a significant influence on the root fresh weight and build-up of new resting spores inside the galls. Generally, as inoculum density increased, root fresh weight and propagation of resting spores increased in clubroot-susceptible cultivar. At the highest levels of inoculum density of *P. brassicae* P1 (10^5^ and 10^7^ resting spores per g soil), no difference was found in either root fresh weight or in the number of new resting spores inside the roots of cv. Visby. The number of propagated resting spores in Visby plants grown in 10^5^ and 10^7^ resting spores per g soil was two- to threefold more than in plants grown in 10^1^ and 10^3^ resting spores per g soil, respectively, at 28 dps. By contrast, the number of new resting spores in cv. Mendel was significantly lower. No significant differences in the propagation of new resting spores were observed in cv. Mendel plants grown in different spore densities of *P. brassicae* P1. In contrast to the less virulent pathotype, growing plants in soil infested with the high virulent *P. brassicae* isolate induced the most rapid increase in root fresh weight, resulting in a statistically significant increase in the number of new resting spores inside the roots. These findings were very similar to our previous study under controlled conditions, where we showed that the first symptoms of clubroot are observed as early as 7 days post-inoculation, and that the severity of symptoms and the root fresh weight of inoculated plants both increased over time and were strongly correlated with higher numbers of pathogen resting spores [25].

Compared with the combination of cv. Visby/*P. brassicae*-P1, the combination of cv. Visby/*P. brassicae*-P1 (+) increased the root fresh weight by 3- and 1.6-fold, and the number of resting spores by 2.5-fold and 1.7-fold, at 10^3^ and 10^7^ resting spores per g soil, respectively. Similarly, the combination of cv. Mendel/*P. brassicae* P1(+) resulted in a significant increase of root weight and the number of propagated resting spores inside the roots.

In connection with another study, trials were conducted to evaluate the effect of growing susceptible and resistant canola cultivars on propagation of *P. brassicae* soil resting spore isolates under mini-plot and field conditions [26]. It was shown that one crop of susceptible canola contributed 1.4 × 10^8^ spores per mL soil in mini-plot experiments and 1×10^10^ spores per g gall under field conditions, and that repeated cropping of susceptible canola resulted in greater root weight compared with the resistant canola lines. 

In the present study, we also observed that both plant growth-parameters and mortality rate were clearly dependent on plant genotype, virulence of the pathotype, and inoculum density*. P. brassicae* isolates induce severe mortality, especially on susceptible oilseed rape cultivar cv. Visby, where the mortality rate was lower at lower spore densities. Similarly, shoot height and shoot fresh weight were significantly decreased in cv. Visby compared with both non-inoculated plants and with inoculated clubroot-resistant cv. Mendel. An increased number of resting spores inside the soil accelerates the decrease in growth parameters in cv. Visby, where this effect was to be more intense when plants were grown in soil infested with the virulent pathotype. Surprisingly, shoot height and shoot fresh weight in cv. Mendel were unaffected by either virulence of the pathogen or the density of resting spores inside the soil. The majority of cv. Mendel plants, grown in either *P. brassicae* P1 soil or in *P. brassicae* P1(+) soil, survived until the last assessment date at 56 dps. The shoot fresh weight increased in the surviving plants relative to non-inoculated ones, probably due to minimized competition between plant genotypes after the death of susceptible plants in neighboring rows.

In conclusion, our results have practical indications for strategies of clubroot management in oilseed rape cultivations, including the development and use of resistant cultivars and cultural practices. We have observed that, on one hand, lower inoculum concentrations of high virulence isolate accelerate the expression of clubbing symptoms and, on the other hand, high inoculum densities of the less virulent pathogen can overcome resistance in resistant cultivar. It should, therefore, be taken into consideration that repeated cultivation of clubroot-resistant cultivars could select for and increase the high virulent resting spores inside the soil.

## 4. Materials and Methods 

All experiments in the present study were conducted twice, where each repetition is referred to as a run. Analysis of variance of common treatments did not show significant differences (*p* ≤ 0.05) between these runs, so the data for analysis and presented in this study were pooled.

### 4.1. Plant and Pathogen Material 

The clubroot-resistant winter oilseed rape cv. Mendel and the clubroot-susceptible cv. Visby used in this study were selected from the German Plant Variety Catalogue in 2016. Both cultivars were obtained from Norddeutsche Pflanzenzucht Hans-Georg Lembke KG, Insel Poel, Germany. Two field isolates of *P. brassicae* were chosen according to their evaluated virulence [1]; a virulent isolate P1 that was virulent on Visby and avirulent on Mendel and classified as 16/31/12 on ECD set [20] or pathotype 1 according to the system of Somé and co-workers [22], and a virulent isolate P1(+) that could overcome the resistance of the clubroot-resistant cv. Mendel, classified as 17/31/31 on ECD set or pathotype 1, briefly named P1(+), on the differential set of Somé and co-workers [22]. The P1 isolate was collected from a naturally infested oilseed rape field in Schleswig-Holstein, Germany in 2012 and the P1(+) isolate originated from a field in Bavaria, Germany in 2013 [1]. Both isolates were preserved as frozen root galls at −20 °C and used for inoculum preparation as needed.

### 4.2. Soil Inoculation and Plant Sowing 

Spore suspensions of each isolate were prepared by homogenizing 650 g frozen clubbed root in 800 mL sterile deionized water in a blender (Vital Mixer Pro, Hollenstedt, Germany) for 10 min at 20,000 rpm. The solution was filtered several times through fine layers of cheesecloth until the suspension was almost free from plant debris. The concentration of the spore suspension was determined with a Fuchs Rosenthal haemocytometer (Hecht-Assistent, Sondheim vor der Rhön, Germany) under a microscope. The greenhouse assays were conducted using large greenhouse trays (300 × 100 × 25 cm), each containing 160 kg mix of potting soil, sand, and peat (5:1:1; pH < 6.5; FloraSelf^®^, Bornheim, Germany). The spore suspension was incorporated into the soil at the appropriate proportion to achieve an inoculum density of 10^1^, 10^3^, 10^5^, and 10^7^ resting spores per g soil. The non-infested control tray was mock inoculated with sterile water. One day after soil inoculation, the seeds of two oilseed rape cultivars were sown in 2 cm deep holes. In total, 34 rows each comprising ten plants were sown. Rows were alternately seeded with seeds of the susceptible cultivar cv. Visby and the resistant cultivar cv. Mendel. The distance between rows and plants within each row was 8.5 cm and 9.5 cm, respectively. Plants were grown under greenhouse conditions at 20 °C, 70% relative humidity, and a 16/8 h day/night regime with a light intensity of 3000 lux for eight weeks. Over the course of the experiment, plants were irrigated every other day to maintain soil moisture, but were not water saturated.

### 4.3. Disease Progress Evaluation 

Disease assessment began at 14 days post sowing (dps), followed by three consecutive measurements at intervals of 14 days (14, 28, 42, and 56 dps). At each assessment date, 20 plants per oilseed rape cultivar and per each treatment (infested and non-inoculated control plants) were dug out. The roots were then carefully washed under tap water to remove soil particles, and clubroot severity was visually assessed based on a scale of 0 to 3 (0 = no galling, 1 = a few small galls, 2 = moderate galling, and 3 = severe galling) [34]. Disease severity index (DSI) was calculated for each treatment at each assessment date using the following equation:

(1) $$DSI=\;\frac{\sum^{\text{​}}\left(n\;\times \;0\;+\;n\;\times \;1\;+\;n\;\times \;2\;+\;n\;\times \;3\right)}{N\;\times \;No.classes\;with\;symptoms}\times 100.\;$$

where ‘*n*’ is number of plants in each class; ‘*N*’ is the total number of plants; and the values 0, 1, 2, and 3 represent the respective symptom severity classes. Thus, DSI expresses the mean value of disease severity at any given moment as a proportion of the maximum possible amount of disease.

Later on, total disease severity from sowing date to 56 dps was assessed by calculating the area under the disease progress curve (AUDPC) values according to the following formula developed by Campbell and Madden [35].

(2) $$\sum_{i=1}^{n-1}\frac{y_{i}+y_{i+1}}{2}\;\times \;\left(t_{i+1}-t_{1}\right)$$

in which ‘*n*’ is the number of observations, ‘*i*’ is the time point of observation, ‘*y_i_*’ is the disease severity index at i^th^ observation, and ‘*t_i_*’ is time (days) at the i^th^ observation.

After rating the disease, plants were cut above the hypocotyl to separate the shoot and the root. The plant height (cm), fresh shoot weight (g), and fresh root weight (g) were measured. The relative values and mean values of 20 replicates were calculated according to the following formula:

(3) $$Relative\;value=\frac{measured\;value\;of\;plant\;parameter}{mean\;value\;of\;non-inoculated\;control}\;\times \;100$$

### 4.4. DNA Extraction From Roots and Quantitative PCR Analysis

A quantitative real-time PCR (qPCR) was performed to calculate resting spore ‘equivalents’ inside the infected roots. For DNA-extraction, nine roots of each cultivar and treatment were harvested at each assessment date. These nine roots were divided into three samples each of three roots, lyophilized for 48 h with a freeze dryer (−20 °C, 1 bar; Christ alpha 1-2, Osterode im Harz, Germany), and subsequently crushed with a ball mill (Retsch KG, Haan, Germany) followed by grinding with a mortar. DNA of 50 mg homogenized root samples was isolated by DNA Spin Kit (Macherey-Nagel, Düren, Germany) according to the manufacturer’s instruction. DNA concentration and purity were determined spectrophotometrically by measuring the absorbance at 260 and 280 nm. All DNA samples were adjusted to 2 ng/μL with Kit’s elution buffer. Quantitative PCR was performed using a CFX 384 Real-Time PCR system (Bio-Rad Laboratories, Hercules, USA) in 10 µl reaction volume. SsoAdvanced™ Universal SYBR^®^ Green Supermix (Bio-Rad, Feldkirchen, Germany) was used according to the manufacturers’ recommendations. The reaction was complemented with 1 µL of the DNA-sample (2 ng/µL) and 0.4 µM of each of the diagnostic primers TC2F and TC2R [36]. Three samples of the individual variants were examined, and each DNA-sample was measured in three technical replicates from which the mean starting quantity values were calculated. The PCR program consisted of a 3 min initial denaturation step at 95 °C, followed by 40 cycles of 5 s at 95 °C, 72 s at 72 °C, and 2 min at 72 °C. Fluorescence was detected after each elongation step, and the program was completed with a final elongation step of 2 min at 72 °C. For evaluation of the amplification specificity, melting curve analysis was performed by an initial denaturation step at 95 °C for 10 s, followed by a 55 °C step for 10 s, and subsequent measurements within a range of 65 °C to 95 °C (every 5 s in 0.5 °C temperature increments).

The quantities of *P. brassicae* DNA in roots were calculated using a standard curve, generated from 10-fold serial dilutions of known concentrations of *P. brassicae* DNA ranging from 5 ng/μL to 5 pg/μL. For quantification of *P. brassicae* DNA concentrations inside infected root tissue, a 10-fold dilution series was run on the same PCR plate. For the standard curve, frozen infected galls were cleaned with tap water and sterilized with 70% ethanol for 2 min. After the galls were rinsed with sterile water, they were peeled, and the central clean portion of the roots was homogenized in sterile water with a blender as described above. The pure solution was then transferred to 50 mL Falcon tubes and centrifuged for 20 min (2000× *g*). The upper layer of the pellet containing spores was transferred to a new tube. The pellet was re-suspended in 5 mL of 50% sucrose (Merck KGaA, Darmstadt, Germany) and the suspension was centrifuged for 10 min (2000× *g*). The supernatant was then transferred to a new tube, washed with 30 mL sterile water, and centrifuged for 15 min (2000× *g*). The pellet was dissolved with 5 mL sterile water and treated with 1000 µg/mL streptomycin (Carl Roth GmbH & Co. KG, Karlsruhe, Germany) for 1 h at 37 °C in order to limit bacterial growth. Later, the suspension was centrifuged for 10 min (2000× *g*). The pellet was washed with 5 mL sterile water, centrifuged for 10 min (2000× *g*), and the supernatant was discarded. The pellet was dissolved in 3 mL sterile water and the number of resting spores was determined with a Fuchs Rosenthal haemocytometer (Hecht-Assistant, Sondheim vor der Rhön, Germany) under a light microscope. Total DNA was extracted with DNA Spin Kit (Macherey-Nagel, Düren, Germany) from 1.66 × 10^9^ resting spores.

### 4.5. Statistical Analysis

Prior to analysis of variance, each data set was tested for homogeneity of variance using a normal probability plot. Analysis of variance of the data was performed separately for each experimental greenhouse run. In two experimental runs, no significant effect of repetition was observed. Therefore, the data from these two experimental runs were combined for subsequent analyses. Comparisons between treatments and mean clubroot rating were performed by analysis of variance (ANOVA) using Tukey’s honestly significant difference (HSD) test and were considered significant at *p* ≤ 0.05 in Statistica version 9.1 (Stat Soft, Inc., Tulsa, OK, USA). Correlation coefficient (Spearman rank) was used to analyze the relationship between the inoculum densities in the soil and AUDPC, and between the inoculum densities and the number of propagated resting spores per g gall.

## Figures and Tables

**Table 1:** Total area under disease progress curve (AUDPC) for clubroot disease severity of two winter oilseed rape (OSR) cultivars inoculated with different isolates and inoculum densities of *Plasmodiophora brassicae.*

**OSR Cultivar ^1^**	**Inoculum** **Density**	***P. brassicae*-P1 ^2^**			***P. brassicae*-P1(+) ^3^**		
		AUDPC ^4^	SD ^5^	Stats. ^6^	AUDPC	SD	Stats.
							
Visby	1 × 10^1^	630	±45	bc	1563	±75	cd
	1 × 10^3^	1470	±92	cd	3430	±32	ef
	1 × 10^5^	3162	±206	ef	3477	±16	ef
	1 × 10^7^	3483	±4	ef	3500	±0	ef
Mendel	1 × 10^1^	23	±16	a	624	±48	bc
	1 × 10^3^	88	±61	a	1639	±62	cde
	1 × 10^5^	82	±40	a	2637	±16	e
	1 × 10^7^	327	±49	ab	2911	±20	e

^1^ Clubroot-susceptible oilseed rape cv. Visby and -resistant cv. Mendel; ^2^ *P. brassicae* P1: virulent on cv. Visby and avirulent on cv. Mendel; ^3^ P1(+): virulent on both cultivars; ^4^ area under the disease progress curve; disease assessment started at 14 days post sowing, followed by three consecutive measurements at intervals of 14 days. Data are pooled across two runs (i.e., repetitions). Means of replications ± ^5^ standard deviations of replicates and ^6^ statistical analysis; similar letters imply no significant difference between rows at a level of *p* ≤ 0.05 calculated by Tukey HSD test.

**Table 2:** Spearman’s rank correlation coefficient between soil inoculum densities (10^1^, 10^3^, 10^5^, and 10^7^ spores per g soil) of two pathotypes of *P. brassicae* and area under disease progress curve (AUDPC).

Variable (Cultivar/Pathotype) ^1^	Linear Equation	r_s_	*p*-Value
cv. Visby P1	y = 1024.9x − 376.25	0.95	0.000
cv. Mendel P1	y = 71.228x − 109.73	0.72	0.005
cv. Visby P1(+)	y = 891.33x − 280	0.81	0.006
cv. Mendel P1(+)	y = 785.75x − 11.667	0.89	0.001

^1^ clubroot-susceptible oilseed rape cv. Visby and -resistant cv. Mendel; P1: *P. brassicae* isolate virulent on cv. Visby and avirulent on cv. Mendel; P1(+): virulent on both cultivars; correlation is significant at *p* ≤ 0.05.
